# A prospective comparison of two computer aided detection systems with different false positive rates in colonoscopy

**DOI:** 10.1038/s41746-024-01334-y

**Published:** 2024-12-19

**Authors:** Goh Eun Chung, Jooyoung Lee, Seon Hee Lim, Hae Yeon Kang, Jung Kim, Ji Hyun Song, Sun Young Yang, Ji Min Choi, Ji Yeon Seo, Jung Ho Bae

**Affiliations:** 1 Department of Internal Medicine and Healthcare Research Institute, Healthcare System Gangnam Center, Seoul National University Hospital, Seoul, Korea; 2 Department of Internal Medicine, Seoul National University College of Medicine, Seoul, Korea

**Keywords:** Clinical trial design, Disease prevention

## Abstract

This study evaluated the impact of differing false positive (FP) rates in two computer-aided detection (CADe) systems on the clinical effectiveness of artificial intelligence (AI)-assisted colonoscopy. The primary outcomes were adenoma detection rate (ADR) and adenomas per colonoscopy (APC). The ADR in the control, system A (3.2% FP rate), and system B (0.6% FP rate) groups were 44.3%, 43.4%, and 50.4%, respectively, with system B showing a significantly higher ADR than the control group. The APC for the control, A, and B groups were 0.75, 0.83, and 0.90, respectively, with system B also showing a higher APC than the control. The non-true lesion resection rates were 23.8%, 29.2%, and 21.3%, with system B having the lowest. The system with lower FP rates demonstrated improved ADR and APC without increasing the resection of non-neoplastic lesions. These findings suggest that higher FP rates negatively affect the clinical performance of AI-assisted colonoscopy.

## Introduction

Colorectal cancer (CRC) ranks as the third most common cancer globally and is the second leading cause of cancer-related deaths^1^. The endoscopic detection and resection of precancerous polyp including adenomas are vital for reducing CRC risk, making colonoscopy the gold standard for both diagnosing and preventing CRC^2^. Despite its efficacy, 24–27% of adenomas may be missed during the procedure, significantly contributing to the occurrence of interval CRC^3^. Although advancements in colonoscopy techniques, such as improved bowel preparation, enhanced imaging, and additional endoscopic devices, have been made, there remains considerable variability in adenoma detection rates (ADR) among endoscopists^4^. Given the technical and cognitive limitations associated with standard colonoscopy, the integration of artificial intelligence (AI) into colonoscopy procedures holds great promise.

Computer-aided detection (CADe) have been shown to increase the ADR and reduce the adenoma miss rate in many randomized controlled trials (RCTs)^5–11^. A meta-analysis, encompassing 33 RCTs, revealed that CADe-assisted colonoscopy enhances the relative ADR by 24% and the mean adenomas per colonoscopy (APC) by 39% when compared to standard colonoscopy^12^. However, recent studies examining the practical application of CADe systems in clinical practice have raised concerns about the discrepancy between the expected benefits of CADe demonstrated in previous RCTs and the actual outcomes observed in real-world settings. Those real-world studies have not shown an improved ADR in CADe-assisted colonoscopy compared to the standard colonoscopy. This observation raises a question for the expectation that endoscopist-CADe collaboration invariably leads to better performance.

Several factors can contribute to the variability in the effectiveness of CADe-assisted colonoscopy, including differences in study design, patient population, the proportion of subtle lesions such as ultra-flat lesions, and the specific CADe device used^12^. Among these multifactorial causes, the frequent occurrence of false positives (FPs) from CADe systems may significantly impact the collaboration between endoscopists and CADe^13^. In a study investigating the performance and attitudes of physicians after using real-time CADe in clinical practice, most physicians expressed concerns regarding the number of FPs, a high level of distraction, and prolonged withdrawal time^14^. An excess number of FPs may reduce the motivation to use CADe, potentially lead to unnecessary polypectomies, and ultimately hinder the widespread implementation of CADe in practice. Currently, addressing FPs is ranked third in importance among future research questions related to CADe^15^.

The number of FPs is an important outcome in numerous CADe studies. However, a significant challenge in these studies is the lack of a uniform definition of FPs, with various studies employing different definitions^16–18^. Additionally, the assessment of FP performance has been conducted using diverse scales, such as per frame, per polyp, or per procedure analysis, and across different datasets^17^. These limitations in the current literature hinder the direct comparison of performance between the systems and accurate evaluation of the clinical impact of FPs in CADe-assisted colonoscopy.

In this study, we sought to investigate the impact of FPs generated by CADe systems on the interactions among endoscopists. We hypothesized that frequent false alarms from CADe systems might lead to distraction or fatigue during routine clinical practice, thereby potentially causing significant alarms to be overlooked. To explore this, we evaluated the polyp detection performance of two investigational CADe systems, both characterized by high sensitivity but differing in their rates of FPs. These devices were assessed using identical benchmarks and by the same group of endoscopists. Additionally, we examined the safety profiles of the two systems according to their false positive performance.

## Results

### Baseline characteristics

The baseline characteristics of the study population are shown in Table 1. A total of 3,047 subjects were included in the study. Among them, 1,591 (52.2%) subjects underwent standard colonoscopy; 763 (25.0%) were assigned to the A system with high FP and 693 (22.7%) were assigned to the B system wit low FP. There were no significant differences in age, sex distribution, or bowel preparation state among the three groups. Withdrawal time and inspection time were significantly longer in both CADe groups than in the control group.

**Table 1:** Baseline characteristics of the study population

	Control (*n* = 1591)	A system (*n* = 763)	B system (*n* = 693)	*P* value
Male, *n* (%)	854 (53.7)	436 (57.1)	404 (58.3)	0.076
Age, years	57.7 ± 8.0	57.4 ± 7.6	56.9 ± 7.3	0.077
BPPS, *n* (%)				0.794
6	120 (7.5)	59 (7.7)	44 (6.3)	
7	66 (4.1)	26 (3.4)	28 (4.0)	
8	160 (10.1)	73 (9.6)	61 (8.8)	
9	1245 (78.3)	605 (79.3)	560 (80.8)	
†WT, (min)	8.5 ± 3.1	9.2 ± 3.6	9.2 ± 3.5	< 0.001
Inspection time, (min)	7.1 ± 2.0	7.6 ± 2.3	7.6 ± 2.3	< 0.001

Continuous variables are presented as mean ± standard deviation and categorical variables are presented as number (%).

AI, Artificial intelligence; BBPS. Boston Bowel Preparation Scale; WT, withdrawal time

†Withdrawal time includes biopsy and polypectomy time.

### Comparison of polyp detection: per-patient analysis

**Fig. 1:**
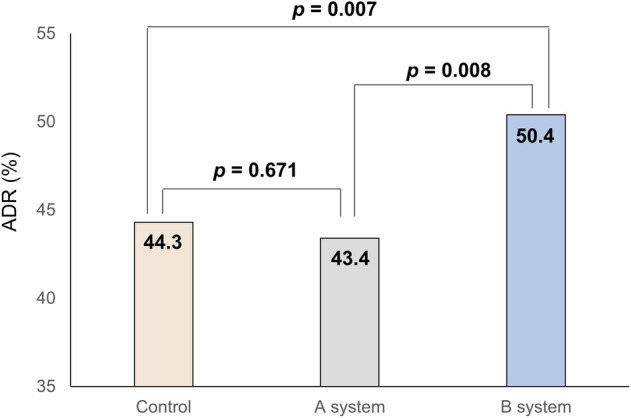
Comparison of the adenoma detection rate, per-patient analysis. The ADR was 44.3% in the control group, 43.4% in the A system group, and 50.4% in the B system group (*p* = 0.012). ADR adenoma detection rate.

As shown in Table 2 and Fig. 1, there was a significant difference in ADR among the three groups (*p* = 0.012). The ADR was 44.3% in the control group, 43.4% in the A system group, and 50.4% in the B system group. Upon adjustment for age, sex, and inspection time, the ADR in the B system was significantly higher compared to the control group (relative risk [RR], 1.11; 95% confidence interval [CI], 1.02–1.22). However, no difference was observed in the ADR in the A system (RR, 0.95; 95% CI, 0.86–1.04). When we compared the two CADe systems, the ADR in the B system was significantly higher compared to the A system (RR, 1.17; 95% CI, 1.05–1.30).

**Table 2:** Adenoma detection rate, per patient analysis

Group	No. of patients	No. with adenomas	Detection rate (%)	Crude	Adjusted
				RR (95% CI)	RR (95% CI)^#^
Control	1591	705	44.3	1 (Ref.)	1 (Ref.)
A system	763	331	43.4	0.98 (0.89, 1.08)	0.95 (0.86, 1.04)
B system	693	349	50.4	1.14 (1.04, 1.25)	1.11 (1.02, 1.22)
B system vs A system				1.16 (1.04, 1.30)	1.17 (1.05, 1.30)

^#^Age, sex, and inspection time adjusted.

*RR* relative risk, *CI* confidence interval.

Next, we stratified the total endoscopists by the endoscopist’s performance according to ADR (above 40% vs below 40%). When we compared the ADR stratified by the endoscopist’s performance, high-performing endoscopists showed no improvement in ADR across both systems and even demonstrated a trend toward decreased ADR in System A (RR, 0.91; 95% CI, 0.81–1.01). For low-performing endoscopists, ADR in System B was significantly higher compared to the control group (RR, 1.22; 95% CI, 1.02–1.45), though there was no additional improvement in System A (Table 3).

**Table 3:** Stratified analysis of adenoma detection rate by endoscopists’ performance, per patient analysis

Group	No. of patients	No. with adenomas	ADR (%)	Crude	Adjusted
				RR (95% CI)	RR (95% CI)^#^
Endoscopists with ADR > 40%					
Control	477	452	48.7	1 (Ref.)	1 (Ref.)
A system	288	252	44.5	0.91 (0.81, 1.01)	0.91 (0.81, 1.01)
B system	996	935	52.2	1.07 (0.96, 1.19)	1.04 (0.94, 1.16)
Endoscopists with ADR < 40%					
Control	409	253	38.2	1 (Ref.)	1 (Ref.)
A system	144	100	41.0	1.07 (0.90, 1.28)	1.01 (0.85, 1.21)
B system	113	97	46.2	1.10 (1.01, 1.44)	1.22 (1.02, 1.45)

^#^Age, sex, and inspection time adjusted.

*ADR* adenoma detection rate, *RR* relative risk, *CI* confidence interval.

We also compared ADR between the two CADe systems, each with different FP rates, according to polyp characteristics, including morphology, size, and location (Table 4). The ADR of System B was higher than that of standard colonoscopy for detecting non-polypoid lesions (RR, 1.15; 95% CI, 1.03–1.29), lesions smaller than 10 mm (RR 1.10; 95% CI, 1.01–1.21), and lesions located in the proximal colon (RR 1.14; 95% CI, 1.03–1.26). When comparing the two CADe systems, significant differences were found in the subgroups with non-polypoid adenomas, adenomas with <10 mm, in the proximal adenomas. Additional information on sessile serrated lesion and polyp detection was described in Supplementary Tables 1-2 and Supplementary Fig. 1.

**Table 4:** Adenoma detection rate by polyp characteristics, per-patients analysis

	A system vs control RR (95% CI)^#^	*P*-value	B system vs control RR (95% CI)^#^	*P*-value	B system vs A system RR (95% CI)^#^	*P*-value
Total	0.95 (0.86, 1.04)	0.29	1.11 (1.02, 1.22)	0.022	1.17 (1.05, 1.30)	0.004
Morphology						
Polypoid	0.97 (0.80, 1.18)	0.777	1.12 (0.92, 1.35)	0.258	1.15 (0.92, 1.43)	0.221
Non-polypoid	0.96 (0.85, 1.08)	0.476	1.15 (1.03, 1.29)	0.017	1.20 (1.05, 1.38)	0.007
Size						
< 10 mm	0.95 (0.86, 1.05)	0.296	1.10 (1.01, 1.21)	0.035	1.16 (1.04, 1.30)	0.006
≥ 10 mm	0.93 (0.47, 1.83)	0.827	1.53 (0.83, 2.83)	0.176	1.65 (0.79, 3.44)	0.181
Location						
Proximal colon^a^	0.97 (0.87, 1.08)	0.566	1.14 (1.03, 1.26)	0.014	1.18 (1.04, 1.33)	0.009
Distal colon^b^	0.95 (0.75, 1.20)	0.666	1.09 (0.86, 1.39)	0.458	1.15 (0.87, 1.52)	0.314

*RR* relative risk, *CI* confidence interval.

^#^ After adjustment for age, sex and inspection time

^a^ Cecum, ascending, and transverse.

^b^ Descending, sigmoid, and rectum.

### Comparison of polyp detection: per polyp analysis

**Fig. 2:**
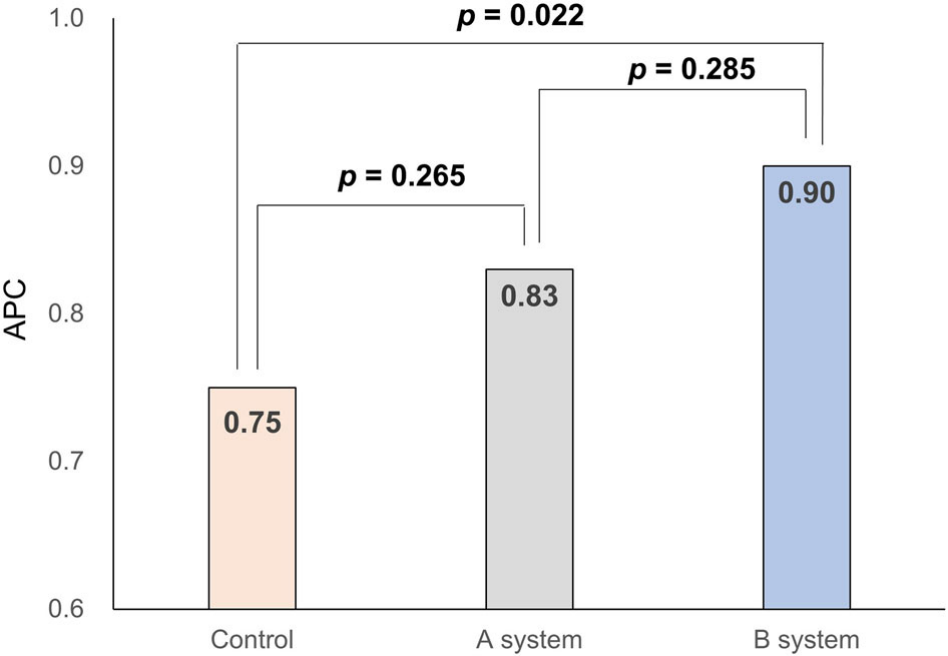
Mean number of adenomas per colonoscopy. The mean APCs of the control group, A system group, and B system group were 0.75, 0.83, and 0.90, respectively. APC adenomas per colonoscopy.

The mean APCs of the control group, A system group, and B system group were 0.75, 0.83, and 0.90, respectively (Fig. 2). After adjustment for age, sex, and inspection time, the difference in APC among the three groups is shown in Supplementary Table 3. The mean number of APCs was significantly higher in the B system compared to the control group (incidence rate ratio, 1.14, 95% CI, 1.01–1.29). The B system also showed higher APC for non-polypoid lesions (IRR, 1.21; 95% CI, 1.04–1.41) and lesions located in the proximal colon (RR 1.16; 95% CI, 1.00–1.34). When we compared the mean APC between the two CADe systems, a statistically significant increase in APC was found for nonpolypoid lesions in the B system compared to the A system. In a stratified analysis by the endoscopist’s performance, the mean APC was significantly higher in the B system compared to the control group in low-performing endoscopists (Supplementary Figure 2). The mean number of polyps per colonoscopy by polyp characteristics are shown in Supplementary Table 4.

### Safety analysis

**Fig. 3:**
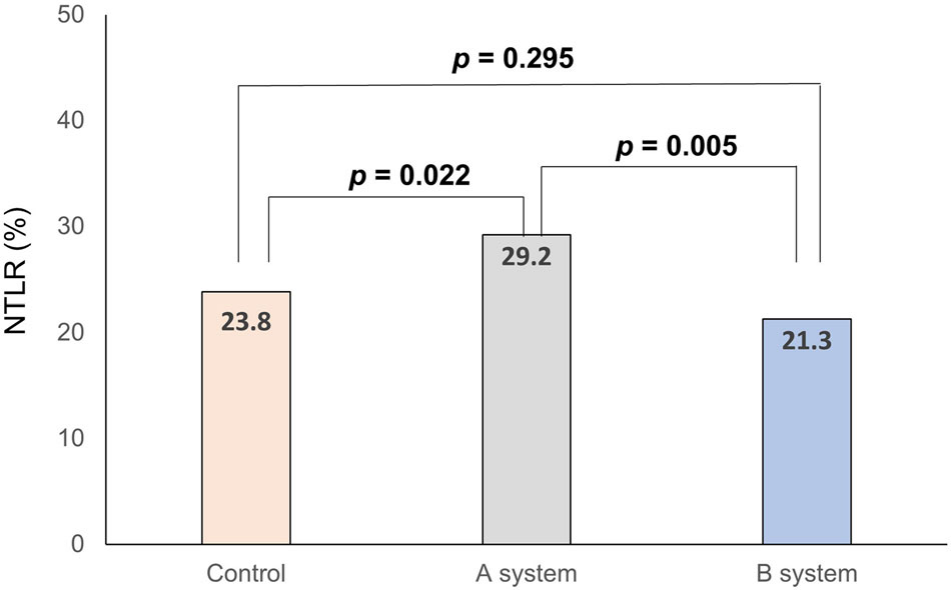
Comparison of non-true lesion resection rate, per-patient analysis. The NTLR was 23.8% in the control group (239 of 1,003 patients), 29.2% (147 of 503 patients) in the A system group, and 21.3% (101 of 473 patients) in the B system group. NTLR, non-true lesion resection rate.

To evaluate the safety effect of FPs on the performance of CADe systems, we compared the non-true lesion resection rate (NTLR) among three groups. The NTLR was 23.8% in the control group (239 of 1003 patients), 29.2% (147 of 503 patients) in the A system group, and 21.3% (101 of 473 patients) in the B system group (Fig. 3). After adjusting for age, sex, and inspection time, there was a 26% increase in NTLR in the A system group (RR, 1.26; 95% CI, 1.06–1.49) compared to the control group while there were no significant differences in the NTLR in the B system group (RR, 0.90; 95% CI, 0.74–1.11, Table 5). When we compared the two CADe systems, the NTLR was significantly decreased in the B system compared to the A system (RR, 0.72; 95% CI, 0.58–0.89). In the stratified analysis by endoscopist’s performance, the NTLR in system A was higher compared to the control group among high-performing endoscopists (RR, 1.35; 95% CI, 1.09–1.67) (Table 6).

**Table 5:** Non-true lesion resection rate, per patient analysis

Group	No. of patients	No. of patients with non-true lesions	NTLR (%)	Crude	Adjusted
				RR (95% CI)	RR (95% CI) ^#^
Control	1003	239	23.8	1 (Ref.)	1 (Ref.)
A system	503	147	29.2	1.23 (1.03, 1.46)	1.26 (1.06, 1.49)
B system	473	101	21.3	0.90 (0.73, 1.10)	0.90 (0.74, 1.11)
B system vs A system				0.73 (0.59, 0.91)	0.72 (0.58, 0.89)

^#^Age, sex, and inspection time adjusted.

*NTLR* Non-true lesion resection rate, *RR* relative risk, *CI* confidence interval.

**Table 6:** Stratified analysis of non-true lesion resection rate, per patient analysis

Group	No. of patients	No. of patients with non-true lesions	NTLR (%)	Crude	Adjusted
				RR (95% CI)	RR (95% CI)^#^
Endoscopists with ADR > 40%					
Control	637	144	22.6	1 (Ref.)	1 (Ref.)
A system	356	107	30.1	1.33 (1.07, 1.65)	1.35 (1.09, 1.67)
B system	340	71	20.39	0.92 (0.72, 1.19)	0.94 (0.74, 1.21)
Endoscopists with ADR < 40%					
Control	366	95	26.0	1 (Ref.)	1 (Ref.)
A system	147	40	27.2	1.05 (0.76, 1.44)	1.06 (0.77, 1.47)
B system	133	30	22.6	0.87 (0.61, 1.24)	0.84 (0.59, 1.21)

^#^Age, sex, and inspection time adjusted.

NTLR, non-true lesion resection rate; RR, relative risk; CI, confidence interval.

When we compared the mean number of non-true lesions per colonoscopy (NPC) among the three groups, there was no statistical significance in the differences in the mean number of non-true lesions among the three groups (0.50 in the control, 0.54 in the A system, 0.53 in the B system group, Supplementary Fig. 3).

## Discussion

This large prospective study included approximately 3000 patients and yielded the first comparison of two real-time CADe systems with different FP rates for colonoscopy in daily clinical practice among endoscopists with a high baseline ADR. The results showed notable variability in the ADR and APC in the CADe groups compared to the control group, dependent on the specific system in use. While both CADe systems exhibited high standalone performance with 100% per-lesion sensitivity and an excellent reaction time of less than 1 s, a significant improvement in the ADR and APC was only found in the B system that displayed a lower FP rate at 0.6%, compared with the control. Our study demonstrates that a higher FP occurrence in colonoscopies could adversely affect the clinical effectiveness of AI-assisted colonoscopy in practice.

Although FPs in CADe systems have been identified as a potential barrier to their clinical adoption, the precise prevalence and clinical impact of these FPs remained unclear. Recently, Troya et al. conducted a direct comparison of the performance across three commercial CADe systems, utilizing the same dataset and conditions^19^. Their research revealed that all three systems demonstrated a polyp sensitivity exceeding 98%, although the FP rates exhibited significant variability, ranging from 0.63% to 3.80%, contingent upon the system’s brand, version, and operational mode. This variability in FP rates underscores the need for standardized testing protocols and criteria to accurately assess and compare the efficacy of different CADe systems, ensuring that their integration into clinical practice does not compromise the quality of colonoscopy.

Other studies examined the prevalence of FPs and their clinical relevance using a systematic method and found that FPs occurred at a rate of approximately one per every 30 s of withdrawal time, totaling 27 FPs per colonoscopy, predominantly due to artifacts originating from the bowel wall^13,20^. Those studies concluded that FPs in CADe-assisted colonoscopy resulted in a negligible increase of 1% in the total withdrawal time, equating to 1.6 relevant FPs per colonoscopy. However, those studies were a post-hoc analysis of a prior CADe RCT, and thus did not aim to evaluate the impact of FPs on polyp detection rates.

Of the two CADe models in this study, system A produced more FPs, occurring 2.5 times per lesion and 5 times per frame. However, beyond these quantitative differences in FP frequency, qualitative differences in FP duration (system A produce 10 times more FP in 0.1–1 s duration) between the two systems may have significantly influenced the clinical effectiveness of AI-assisted colonoscopy. Bounding box durations shorter than an endoscopist’s reaction time are likely to be overlooked during CADe-assisted colonoscopy. According to the American Society for Gastrointestinal Endoscopy’s recommendations for AI-assisted endoscopy, endoscopists require at least 1 to 2 s to classify lesions from a histological perspective^21^. However, visual processing time from the eye to the brain, such as when adjusting motor functions (e.g., while driving), takes approximately 0.1 to 0.15 s^22^. These differences suggest that the response time needed to recognize the presence of a lesion is shorter than the time required to assess its characteristics.

When the bounding box duration exceeds 0.1 to 0.15 s, endoscopists are more likely to focus on discerning whether the lesion is true or false, raising an important point regarding the impact of “alarm fatigue”. FPs lasting less than 0.1 s are unlikely to draw significant attention from the endoscopist, whereas longer-lasting FPs may more substantially affect their focus and decision-making process. Although this study does not specifically aim to explore the clinical significance of differences in FP duration, we believe that qualitative differences in FP duration between the two CADe systems may be a critical factor influencing clinical outcomes.

The phenomenon known as the “crying wolf effect” describes a phenomenon wherein repeated false alarms or warnings result in the diminution of attention to authentic alerts^23–25^. Frequent false alarms contribute to the development of alarm fatigue, which has been shown to increase healthcare providers’ physical fatigue, workload, and decrease their concentration^26^. Clinical alarm fatigue results in clinicians becoming desensitized to alerts, causing them to ignore or silence alarms, thereby increasing the risk of overlooking critical clinical conditions^27^. A study reported that 80% of alarm signals generated during cardiac surgeries did not lead to any therapeutic intervention, highlighting the need for advancements in alarm system technology to enhance patient monitoring and safety in surgical settings^23^. These non-actionable or avoidable alarms might cause physician to be desensitized and affect their responses, leading to delayed or no responses^28^. Emergency care research Institute reported numerous alarm-related incidents, including alarms being ignored, or missed, and alarm fatigue was listed as a major health hazard^29^. The Food and Drug Administration has reported over 500 alarm-related patient deaths within the last 5 years^30^. These findings emphasize the critical importance of understanding and managing alarm fatigue in clinical practice. Our study suggests that the ‘crying wolf’ effect may also be evident in the use of colonoscopy with CADe systems. Recurrent FPs can divert the attention of endoscopists, increasing the likelihood of missing true lesions, thereby not only diminishing the overall effectiveness of CADe systems but also potentially compromising patient outcomes and safety.

In recent studies conducted in a real clinical setting, the use of commercialized CADe systems did not result in performance improvement^14,31^, and the results of the survey suggest that the current level of FP activation may not meet the standards for the widespread implementation of CADe by all endoscopists, and that the interactions between AI and individual endoscopists could be more sensitive to FPs^14^. Although no consensus has been reached on an acceptable level of FP occurrence, our experience suggests that fewer than 10 FPs per colonoscopy (irrespective of the duration time in FPs) may facilitate the broader adoption of CADe systems. Further studies are warranted to clarify the optimal balance between sensitivity and FPs.

In additional analysis for adenoma detection, system B showed higher ADR compared to the system A in the detection of non-polypoid lesion, <10 mm in size and located in the proximal adenomas. Conversely, system A showed no differences in ADR or APC across polyp characteristics when compared to standard colonoscopy. These results suggest that the frequent FP occurrence of CADe may have negatively impacted system A’s effectiveness in detecting non-polypoid and proximally located adenomas, which are known contributors to missed lesions in standard colonoscopy^3^. When we conducted a stratified analysis of key outcomes, including ADR and APC based on endoscopists’ performance levels, high-performing endoscopists showed no improvement in ADR across both systems and even demonstrated a trend toward decreased ADR in system A. While ADR in system B was significantly higher compared to the control group, there was no additional improvement in system A in low-performing endoscopists. These results suggest that high-performing endoscopists often encounter a ‘ceiling effect’ in standard colonoscopy, limiting further ADR improvements and that frequent FPs of CADe may negatively affect adenoma detection during AI-assisted colonoscopy.

Concerns have been raised regarding the potential for FPs in CADe systems to precipitate unnecessary polypectomies of non-neoplastic polyps. A retrospective study following the implementation of a CADe system in a real-world setting reported a significant increase in the number of polypectomies performed for hyperplastic polyps compared to a historical control group (1.35 vs. 1.05)^14^. In contrast, an RCT evaluated the safety implications of using a CADe system with a 2.3% FP rate by evaluating true histology rate (THR)^32^. The THR was defined as the total number of histologically confirmed adenomas, sessile serrated lesions, and large (>10 mm) hyperplastic polyps of the proximal colon resected divided by the total number of resected lesions. There was no significant decrease in the THR with the use of the CADe device (standard vs. CADe: 71.7% vs. 67.4%)^32^ The aforementioned studies offer conflicting insights into the safety of CADe systems, concerning the issue of unnecessary polypectomies. Moreover, the absence of direct comparisons focused on the FP performance of CADe systems, coupled with variations in the management of non-neoplastic polyps across institutions or among endoscopists, may hinder the interpretation of these outcomes. In our study, we directly compared NTLR in two CADe systems according to the consistent resection policy, showing that the use of the A system group with a high FP rate resulted in a 26% increase in the polypectomy rate for non-clinically significant lesions compared with the control group. Interestingly, the NTLR in system A was higher than in the control group specifically among high-performing endoscopists. While the exact reason for this is unclear, we speculate that high-performing endoscopists often take a more vigilant approach to inspection, making them more likely to respond to potential lesion alerts, even when these are false positives. This heightened diligence may lead to more unnecessary polypectomies, as they prioritize caution over omission. However, the NPC did not differ significantly among the three groups. These outcomes may hold varied implications for cost-effectiveness analysis across countries with different payment or reimbursement systems.

The strengths of our study include a large sample size, the inclusion of all consecutive patients during the study period, an average-risk patient population, all of which reflect real-world practice. In RCTs, a very controlled selection of patients, procedures, and examiners is implemented to optimize the trial’s ability to test the intervention under ideal circumstances. However, in real-world trials, such optimization is not feasible, and the performance of the intervention is tested as it would be in routine clinical practice. Consequently, the adverse effects of FPs, such as the “crying wolf” effect, may not demonstrate significant clinical importance in previous RCTs with optimal sample sizes and non-consecutive design. However, their impact becomes more pronounced in real-world studies involving high clinical workloads. This discrepancy may explain the differences in the effectiveness of CADe observed between controlled trials and real-world applications.

However, this study also has several limitations. Firstly, the non-randomized nature of the study and the absence of blinding among participating endoscopists to the CADe intervention could potentially introduce bias. Such bias may manifest in behaviors that promote enhanced mucosal visualization or more meticulous lesion evaluation during AI-assisted colonoscopy. This form of researcher bias has been previously suggested as a cause for discrepancies between RCTs and real-world studies^33^. To address this limitation, our study implemented blinding regarding the hypotheses. Intriguingly, despite the full integration of new technology by the participants, our study revealed no significant improvement in both ADR and APC in the CADe group with a high FP rate as compared to the control group. This finding underscores the clinical significance of the impact of FPs. Secondly, given the non-randomized nature of the study, there may be a potential for selection bias in this study. In the baseline characteristics of the study population, there were no significant differences among the three groups except for withdrawal and inspection time. To overcome the confounding effect of inspection time, we adjusted this variable in the multivariable analysis for all major outcomes.

In conclusion, our study demonstrated that only the CADe system with lower FPs improved the ADR and APC without increasing unnecessary polypectomy. These data suggest that the endoscopists’ performance could be negatively influenced by frequent FP alarms during CADe colonoscopy, and the FP rate could serve as a critical determinant for the incorporation of CADe systems into their clinical practice. This sheds light on the need to find the optimal balance between high sensitivity and the reduction of FPs in CADe development. The primary challenge in developing advanced CADe systems lies in achieving high sensitivity for the avoidance of missed lesions while simultaneously minimizing FPs to zero, a crucial factor for their widespread clinical adoption. Moreover, it is essential to provide comprehensive information about the commercial CADe product, coupled with an effective training program for managing FPs. This will bridge the gap during ongoing technical advancements.

## Methods

### Study setting and design

This non-randomized controlled prospective study was conducted from November 2021 to October 2022 at Seoul National University Hospital (SNUH), Healthcare System Gangnam, and involved eight expert endoscopists. The institution is a tertiary healthcare center, conducting comprehensive medical checkups for approximately 30,000 patients per year, and performing an estimated 10,000 annual screening and surveillance colonoscopies. All participating endoscopists were board-certified gastroenterologists with an ADR ranging from 37.9% to 54.8% in the year before the study period. The demographics of the participating endoscopists during the study period are shown in Supplementary Table 5.

The study design incorporated one control group and two CADe groups: the control group performed standard colonoscopy and the others utilized colonoscopy with CADe systems (AI groups) during the study period. Two convolutional neural network-based CADe systems, namely the A system with a high FP rate and B system with a low FP rate, were deployed in the AI group. Briefly, both CADe systems receive digital images from the colonoscope as input and output a green bounding box only when an instance of the target polyp is recognized, as described previously^34^.

Before the commencement of the study, all endoscopists underwent an offline orientation session that provided them with background information on AI-assisted colonoscopy. Additionally, they performed more than 50 colonoscopies using the CADe system in their clinical practice for 2 months. This background knowledge and experience ensured that all participants had sufficient familiarity with the application of AI in colonoscopy during the study period. However, the study hypothesis (to identify the impact of FP occurrence in CADe colonoscopy) were blinded to participants to avoid researcher bias.

### Study patients

**Fig. 4:**
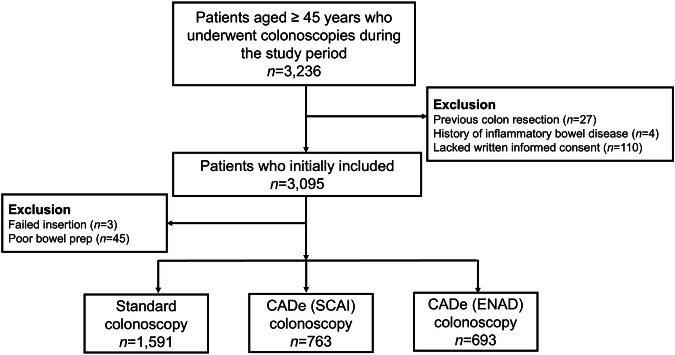
Flow chart of the study population. Among a total of 3,047 subjects, 1,591 (52.2%) subjects underwent standard colonoscopy; 763 (25.0%) were assigned to the A system and 693 (22.7%) were assigned to the B system. CADe, computer-aided detection.

This study enrolled all consecutive individuals aged 45 years or older who underwent colonoscopy for CRC screening or surveillance during the study period. Subjects with a history of CRC, inflammatory bowel disease, or colonic resection were excluded. Additionally, subjects without adequate state of bowel preparation, defined as each segment Boston Bowel Preparation Scale (BBPS) score no less than 2, were excluded from the primary analysis (Fig. 4).

The study protocol was designed with strict adherence to the ethical guidelines outlined in the Declaration of Helsinki and its subsequent amendments. Seoul National University Hospital Institutional review board approval was secured prior to the commencement of the study (approval number: H-2107-235-1240). Written informed consent was obtained from all participating endoscopists and individuals in the AI group. The study was registered at ClinicalTrials.gov (NCT05089071, 2021.11). All authors had access to the study data and reviewed and approved the final manuscript.

### Development and performance of the CADe systems

To develop a CADe system for polyp detection, 15 endoscopists at SNUH Healthcare System Gangnam Center collected colonoscopy image data from more than 10,000 polyps. The two systems were developed by the Division of Bioengineering at SNUH and AINEX corporation. The A system (prototype of ENAD system) was trained using a dataset comprising 197,673 images (158,138 images for the training set and 39,535 images for the internal validation set) derived from 3,121 polyps from the AI database of SNUH Gangnam Center^34,35^. The B system (ENAD 1.0, AINEX corporation, Seoul, Korea) was trained to utilize the same database as the A system, but with a different data composition, which contained 66,397 images (53,118 images for the training set and 13,279 images for the internal validation set) originating from 8,756 polyps. Both systems utilized the YOLOv4 architecture and had the same user interface showing a green box on the screen for polyp detection. To reduce FPs, the B system was trained using only high-quality, well-focused frames, and the system was post-processed using the Kalman filter algorithm. The test set (temporal validation) was composed of new data from examinations that were not included in the training or internal validation sets. The test set consisted of 15,863 images from 80 polyp video clips and 90,144 images from 50 non-polyp clips during withdrawal times. The demographics of the test set were described in Supplementary Table 6.

### Performance of the CADe systems

The performance of both systems in the test set is presented in Table 7. Per-lesion sensitivity was evaluated on a per-polyp basis. It was defined as the number of polyps in more than 20% of polyp frames detected by the CADe system divided by the total number of polyp frames. Per-frame sensitivity was defined as the system’s ability to detect polyps in each frame. The A system achieved a per-lesion sensitivity of 100% and a per-frame sensitivity of 86.4%. The B system achieved a per-lesion sensitivity of 100% and a per-frame sensitivity of 87.1%.

**Table 7:** Performance of the two CADe systems in colonoscopy video test sets

	A system	B system
Sensitivity (per lesion), *n* = 80 polyps	100%	100%
Sensitivity (per frame), *n* = 15,863 frames	86.4%	87.1%
Specificity (per frame), *n* = 90,144 frames	96.5%	99.4%
Reaction time to the first polyp detection	534 ms	1007 ms
False positives		
FP rates (per frame), *n* = 106,007 frames	3.2%	0.6%
Number of false alarms per procedure time	3.28/min	1.24/min
Bowel wall	65.9%	81.0%
Bowel content	34.1%	23.5%
FP numbers according to the bounding box duration	*n* = 164	*n* = 62
≤0.1 s	22 (13.4%)	41 (66.1%)
0.1–1 s	117 (71.3%)	11 (17.7%)
1–2 s	23 (14.1%)	9 (14.5%)
> 2 s	2 (1.2%)	1 (1.7%)

*FP* false positive.

For real-time evaluation, we measured the reaction time for polyp detection, defined as the time interval between the initial appearance of the polyp and the moment when the AI’s detection box accurately identified its location. The reaction time for the A system (534 ms) was shorter than that of the B system (1007 ms).

FPs in this study were defined as any activation of the bounding box that does not correspond to colorectal lesions (both protruded and flat polyps) irrespective of duration and follow the FP causes of NOISE classification^20^. Both an expert endoscopist and a designated annotator reviewed the test video set to identify false activations, and any discrepancies were resolved by consensus with a third expert endoscopist. Multiple FP alerts triggered by the same were counted as a single FP. The FP rate was higher in the A system at 3.2% compared to the B system at 0.6%. The number of FPs per minute in the 50-min video set was 3.28 for the A system and 1.24 for the B system. The total number of FPs generated by the CADe system was 164 in system A and 62 in system B, meaning that system A produced 2.6 times more FPs than system B in the test set. When we further analyzed FPs by its durations, a significant discrepancy was observed in the 0.1–1 s interval, with 117 FPs in system A and only 11 FPs in system B. Most of FPs in both systems occurred for less than 1 s (Table 7 and Supplementary Movie 1).

### Colonoscopy procedures with or without CADe

All colonoscopies were performed using high-definition colonoscopy equipment (CF-HQ290; Olympus Co, Ltd., Tokyo, Japan). Among the twelve available endoscopy rooms, six were equipped with the CADe system. Colonoscopies conducted in rooms with the CADe system were categorized into the AI group. Because this study was conducted during real-world clinical practice rather than a randomized controlled trial, endoscopists could not be assigned to the control and AI groups according to their experience levels. However, all endoscopists operated both with and without the two CADe systems almost evenly according to routine schedule during the designated period.

The use of any additional devices during the procedures was strictly prohibited. Bowel preparation was evaluated and graded using the BBPS by an endoscopist during colonoscopy^36^. During the colonoscopy procedure, all polyps were detected and removed according to routine daily practice as described in detail by Bae et al. and were subsequently evaluated by a board-certified pathologist at Seoul National University Hospital^37,38^. All polyps were classified according to their morphology, size, and location according to the Paris classification^39^.

### Study outcomes

The primary outcome was the comparison of ADR and APC among the control and two CADe groups. ADR was defined as the proportion of patients with at least one histologically proven adenoma. APC was defined as the total number of adenomas divided by the number of colonoscopies performed.

The secondary outcomes included the NTLR, defined as patients with no clinically significant histology on resected lesions divided by the total number of patients with polyp resection; and the number of NPC, defined as the total number of polyps minus the number of polyps with clinically significant histology divided by the total number of colonoscopies. Clinically significant histology included any adenoma, sessile serrated lesion, any hyperplastic polyps ≥10 mm, and proximal hyperplastic polyps ≥5 mm. Other secondary outcomes included the detection rate of sessile serrated lesions, the polyp detection rate, defined as the percentage of colonoscopies in which one or more polyps are detected, and the mean number of polyps per colonoscopy, defined as the total number of polyps detected divided by the total number of colonoscopies performed.

### Sample size and statistical analysis

The sample size for the unconditional comparison of the two study groups relative to a control group adjusted for multiple comparisons was estimated. In the case of ADR, assuming an effect size of 8.4% with the CADe group at 41.4% compared to the control group at 33.0%^10^, while controlling for a Bonferroni correction, with a type I error of 2.5% and a power of 80%, the sample size was calculated as 629 subjects for each group using a two independent proportion test adjusting for multiple comparisons. In the case of APC, we calculated the pooled standard deviation as 1.12 for the control group and 1.37 for the CADe group^10^. With an expected difference in mean scores of 0.22, a standard deviation of 1.12 for the control group and 1.37 for the CADe group, while controlling for a Bonferroni correction, with a type I error of 2.5% and a power of 80%, a sample size of 616 subjects for each group was estimated. Using a conservative approach, the decision was made to opt for the larger number, 629, among 629 and 616.

Categorical variables are described as frequency counts and percentages. Continuous variables are described as mean and standard deviations. Analysis of variance was used to compare variables among groups. Multivariable estimations of prevalence ratios were obtained using log-binomial regression; adjustments were made for age, sex, and inspection time (withdrawal time without polypectomy time). Differences among groups were expressed as the RR with the 95% CI. A Poisson regression model was used to compare the detection of all adenomas (per-polyp analysis). In addition, we conducted a stratified analysis of key outcomes, including ADR, APC, NTLR based on endoscopists’ performance levels to examine more precisely the impact of FPs on endoscopists’ performance. We categorized the 8 endoscopists by ADR (above 40%: range 43.5–58.5% vs. below 40%: range 35.7–39.6%). A *P* value < 0.05 was considered statistically significant. All statistical analyses were performed using R software version 3.5.1.

## Supplementary information

Supplementary Materials

Supplementary Movie 1

## Data Availability

The data, analytic methods, and study materials used in this study will be made available to other researchers upon proper request. Researchers interested in accessing these materials should contact the corresponding author for further information and conditions.
