# Supplementary material for: A prospective comparison of two computer aided detection systems with different false positive rates in colonoscopy
Source: NPJ Digit Med. 2024 Dec 19;7:366. doi: 10.1038/s41746-024-01334-y (PMC11659580; doi:10.1038/s41746-024-01334-y)
Supplement: Supplementary file 1 — Supplementary Materials [file 41746_2024_1334_MOESM1_ESM.pdf]

Supplementary Table 1. Sessile serrated lesion detection rate, per patient analysis.

Group	No. of patients	No. with SSLs	Detection rate (%)	Crude	Adjusted
				RR (95% CI)	RR (95% CI) #
Control	1,591	78	4.9	1 (Ref.)	1 (Ref.)
A system	763	37	4.8	0.99 (0.68, 1.45)	0.94 (0.64, 1.39)
B system	693	39	5.6	1.15 (0.79, 1.67)	1.09 (0.75, 1.60)
B system vs A system				1.16 (0.75, 1.80)	1.16 (0.75, 1.80)

#Age, sex, and inspection time adjusted.

SSL, sessile serrated lesion; RR, relative risk; CI, confidence interval.

Supplementary Table 2. Polyp detection rate, per patient analysis.

Group	No. of patients	No. with polyps	Detection rate (%)	Crude	Adjusted
				RR (95% CI)	RR (95% CI) [#]
Control	1,591	1,003	63	1 (Ref.)	1 (Ref.)
A system	763	503	65.9	1.05 (0.98, 1.11)	1.02 (0.96, 1.09)
B system	693	473	68.3	1.08 (1.02, 1.15)	1.06 (1.00, 1.13)
B system vs A system				1.04 (0.96, 1.11)	1.04 (0.97, 1.11)

[#]Age, sex, and inspection time adjusted.

RR, relative risk; CI, confidence interval.

Supplementary Table 3. Mean number of APC by polyp characteristics, per-polyp analysis.

	Control	A system	B system	A system vs control	B system vs control	B system vs A system
	APC (SD)	APC (SD)	APC (SD)	IRR (95% CI)[#]	IRR (95% CI)[#]	IRR (95% CI)[#]
Total	0.75 (1.16)	0.83 (1.41)	0.90 (1.25)	1.05 (0.92, 1.19)	1.14 (1.01, 1.29)	1.09 (0.94, 1.27)
Morphology						
Polypoid	0.25 (0.77)	0.29 (1.00)	0.26 (0.79)	1.19 (0.92, 1.53)	1.07 (0.83, 1.38)	0.90 (0.67, 1.22)
Non-polypoid	0.50 (1.00)	0.54 (1.14)	0.65 (1.12)	1.00 (0.86, 1.18)	1.21 (1.04, 1.41)	1.22 (1.01, 1.46)
Size						
< 10 mm	0.72 (1.15)	0.79 (1.33)	0.84 (1.17)	1.03 (0.91, 1.18)	1.12 (0.99, 1.27)	1.08 (0.93, 1.26)
≥ 10 mm	0.03 (0.26)	0.04 (0.53)	0.06 (0.53)	1.27 (0.54, 3.01)	1.58 (0.66, 3.75)	1.24 (0.41, 3.79)
Location						
Proximal colon[a]	0.60 (1.11)	0.67 (1.30)	0.75 (1.23)	1.06 (0.91, 1.23)	1.16 (1.00, 1.34)	1.09 (0.92, 1.30)
Distal colon[b]	0.15 (0.54)	0.16 (0.72)	0.16 (0.54)	1.07 (0.92, 1.19)	1.12 (0.84, 1.50)	1.05 (0.27, 1.544)

APC, adenomas per colonoscopy; SD, standard deviation; IRR, incidence risk ratio; CI, confidence interval.

[#] After adjustment for age, sex, and inspection time

[a] Cecum, ascending, and transverse.

[b] Descending, sigmoid, and rectum.

Supplementary Table 4. Mean number of polyps per colonoscopy by polyp characteristics, per-polyp analysis.

	Control	A system	B system	A system vs control	B system vs control	B system vs A system
	PPC (SD)	PPC (SD)	PPC (SD)	IRR (95% CI)[#]	IRR (95% CI)[#]	IRR (95% CI)[#]
Total	1.33 (1.58)	1.45 (1.76)	1.53 (1.62)	1.02 (0.93, 1.12)	1.08 (0.98, 1.18)	1.06 (0.95, 1.18)
Morphology						
Polypoid	0.38 (1.07)	0.42 (1.33)	0.39 (1.07)	1.12 (0.90, 1.4)	1.05 (0.84, 1.31)	0.94 (0.72, 1.22)
Non-polypoid	0.95 (1.44)	1.03 (1.49)	1.14 (1.54)	0.99 (0.88, 1.11)	1.12 (1.01, 1.25)	1.13 (1.00, 1.29)
Size						
< 10 mm	1.27 (1.57)	1.38 (1.65)	1.44 (1.58)	1.01 (0.92, 1.11)	1.08 (0.98, 1.18)	1.06 (0.95, 1.19)
≥ 10 mm	0.06 (0.41)	0.07 (0.76)	0.09 (0.62)	1.10 (0.54, 2.21)	1.19 (0.59, 2.38)	1.08 (0.44, 2.68)
Location						
Proximal colon[a]	1.05 (1.55)	1.12 (1.65)	1.26 (1.64)	1.01 (0.9, 1.13)	1.11 (1.00, 1.24)	1.1 (0.97, 1.25)
Distal colon[b]	0.28 (0.82)	0.33 (1.05)	0.27 (0.79)	1.14 (0.9, 1.44)	1.03 (0.81, 1.31)	0.90 (0.68, 1.21)

PPC, polyps per colonoscopy; SD, standard deviation; IRR, incidence risk ratio; CI, confidence interval.

[#] After adjustment for age, sex, and inspection time

[a] Cecum, ascending, and transverse.

[b] Descending, sigmoid, and rectum.

Supplementary Table 5. Demographics of participating endoscopists.

	Endoscopy experience (yr)	Colonoscopy volume	Familiarity with NICE classification#	Familiarity with WASP classification#	Baseline ADR (%)
A	≥10	≥5,000	High	High	58.5
B	≥10	≥5,000	High	High	52.9
C	≥10	≥5,000	High	High	45.4
D	<10	≥5,000	High	High	43.9
E	<10	<5,000	High	Low	43.5
F	<10	<5,000	High	Low	39.6
G	<10	<5,000	High	Low	39.5
H	<10	<5,000	High	Low	35.7

ADR, adenoma detection rate; NICE, narrow-band imaging international colorectal endoscopic; WASP, Workgroup serrAted polypS and Polyposis

Familiarity with NICE and WASP classification (High/Low) was based on participation in a previous optical diagnosis study.[38]

Supplementary Table 6. Demographics of polyps in the temporal validation set.

Characteristics	n (%)
**Size**	
Diminutive (≤ 5mm)	54 (68)
Small (6–9mm)	22 (28)
Large (≥ 10mm)	4 (5)
**Location**	
Cecum	5 (6)
Ascending	8 (10)
Transverse	38 (48)
Descending	4 (5)
Rectosigmoid	25 (31)
**Appearance**	
Flat	57 (71)
Protruded	23 (29)
**Histopathology**	
Adenoma	50 (63)
Hyperplastic polyp	17 (21)
Sessile serrated lesion	13 (16)

Supplementary Figure 1. Comparison of sessile serrated lesion detection rate, per-patient analysis.

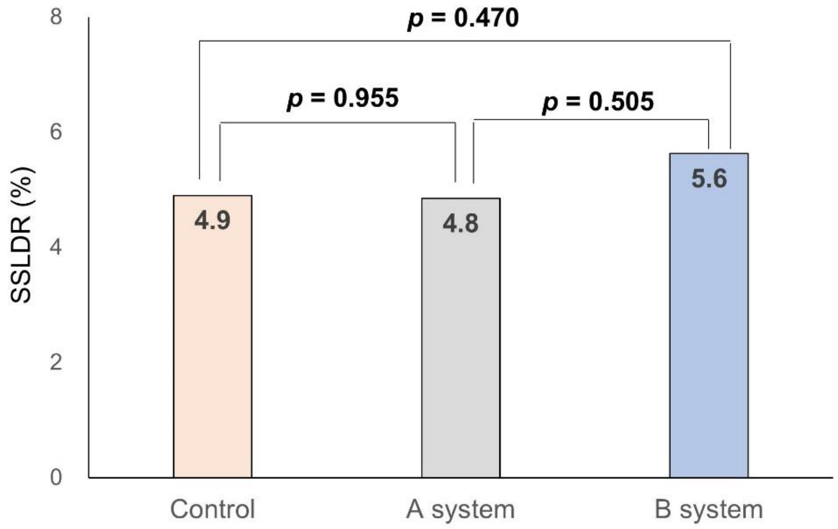

There was no significant difference in sessile serrated lesion detection rate among the three groups

SSLDR, sessile serrated lesion detection rate

Supplementary Figure 2. Mean number of adenomas per colonoscopy by endoscopists' performance.

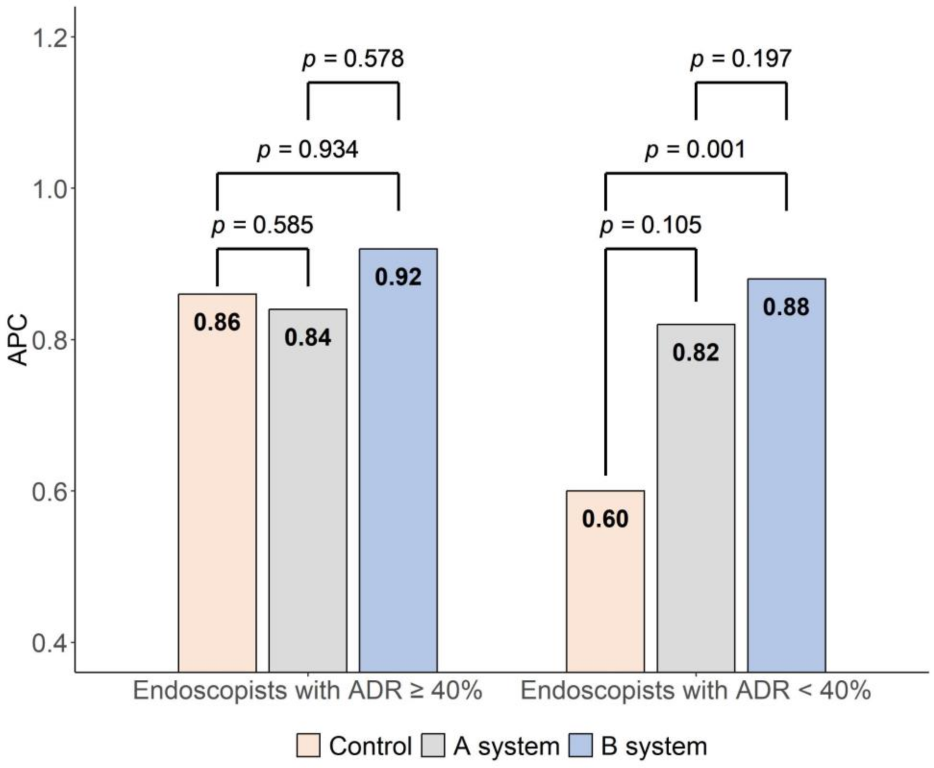

The mean APC was significantly higher in the B system compared to the control group in low-performing endoscopists

APC, adenomas per colonoscopy; ADR, adenoma detection rate

Supplementary Figure 3. Comparison of non-true lesion per colonoscopy.

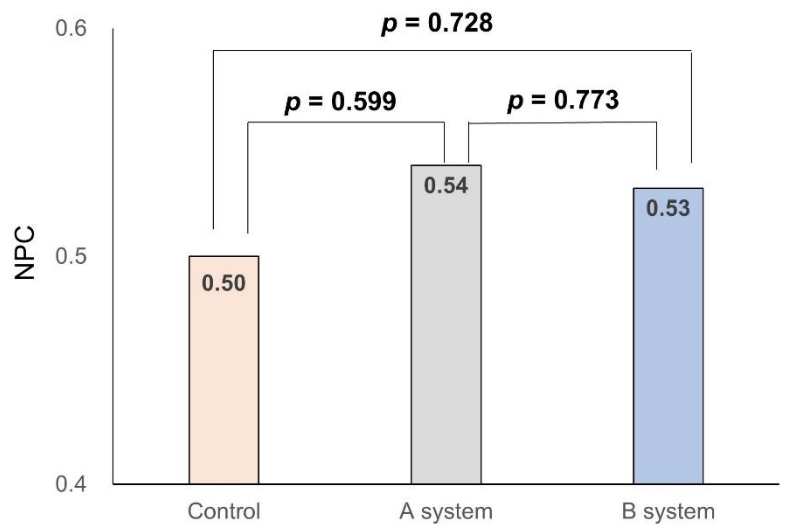

There was no statistical significance in the differences in the mean number of non-true lesions among the three groups.

NPC, non-true lesion per colonoscopy

Supplementary Movie 1. Incidence of false positives by systems A (left) and B (right) in the detection of a sessile serrated lesion. In this video, both systems detected a small-sized sessile serrated lesion. System A demonstrated a slightly faster reaction time and higher per-frame sensitivity compared to system B. However, system A exhibited four false positives (FPs): Three FPs lasting less than 0.1 second, unlikely to draw the endoscopist's attention. However, one FPs lasted more than 0.1 second (7 frame), making them more noticeable to the endoscopist. This FP was caused by the system misinterpreting normal colon structures such as lymphoid follicle, which could lead to unnecessary distractions for endoscopists.
